# Novel Bioactive Nanocomposites Containing Calcium Fluoride and Calcium Phosphate with Antibacterial and Low-Shrinkage-Stress Capabilities to Inhibit Dental Caries

**DOI:** 10.3390/bioengineering10090991

**Published:** 2023-08-22

**Authors:** Abdullah Alhussein, Rashed Alsahafi, Abdulrahman A. Balhaddad, Lamia Mokeem, Abraham Schneider, Mary-Ann Jabra-Rizk, Radi Masri, Gary D. Hack, Thomas W. Oates, Jirun Sun, Michael D. Weir, Hockin H. K. Xu

**Affiliations:** 1 PhD Program in Dental Biomedical Sciences, University of Maryland School of Dentistry, Baltimore, MD 21201, USA; 2 Department of Restorative Dental Sciences, College of Dentistry, King Saud University, Riyadh 11451, Saudi Arabia; 3 Department of Restorative Dental Sciences, Umm Al-Qura University, College of Dentistry, Makkah 24211, Saudi Arabia; 4 Department of Restorative Dental Sciences, College of Dentistry, Imam Abdulrahman Bin Faisal University, Dammam 31441, Saudi Arabia; 5 Department of Oncology and Diagnostic Sciences, University of Maryland School of Dentistry, Baltimore, MD 21201, USA; 6 Department of Advanced Oral Sciences and Therapeutics, University of Maryland School of Dentistry, Baltimore, MD 21201, USA; 7 The Forsyth Institute, Harvard School of Dental Medicine Affiliate, Cambridge, MA 02142, USA; 8 Center for Stem Cell Biology & Regenerative Medicine, University of Maryland School of Medicine, Baltimore, MD 21201, USA; 9 Marlene and Stewart Greenebaum Cancer Center, University of Maryland School of Medicine, Baltimore, MD 21201, USA

**Keywords:** low polymerization stress, nanocomposite, remineralization, bio-interactive, antibiofilm, oral biofilms, caries

## Abstract

Objectives: Composites are commonly used for tooth restorations, but recurrent caries often lead to restoration failures due to polymerization shrinkage-stress-induced marginal leakage. The aims of this research were to: (1) develop novel low-shrinkage-stress (L.S.S.) nanocomposites containing dimethylaminododecyl methacrylate (DMADDM) with nanoparticles of calcium fluoride (nCaF_2_) or amorphous calcium phosphate (NACP) for remineralization; (2) investigate antibacterial and cytocompatibility properties. Methods: Nanocomposites were made by mixing triethylene glycol divinylbenzyl ether with urethane dimethacrylate containing 3% DMADDM, 20% nCaF_2_, and 20% NACP. Flexural strength, elastic modulus, antibacterial properties against *Streptococcus mutans* biofilms, and cytotoxicity against human gingival fibroblasts and dental pulp stem cells were tested. Results: Nanocomposites with DMADDM and nCaF_2_ or NACP had flexural strengths matching commercial composite control without bioactivity. The new nanocomposite provided potent antibacterial properties, reducing biofilm CFU by 6 logs, and reducing lactic acid synthesis and metabolic function of biofilms by 90%, compared to controls (*p* < 0.05). The new nanocomposites produced excellent cell viability matching commercial control (*p* > 0.05). Conclusions: Bioactive L.S.S. antibacterial nanocomposites with nCaF_2_ and NACP had excellent bioactivity without compromising mechanical and cytocompatible properties. The new nanocomposites are promising for a wide range of dental restorations by improving marginal integrity by reducing shrinkage stress, defending tooth structures, and minimizing cariogenic biofilms.

## 1. Introduction

Methacrylate-derived composites are the predominant option to restore tooth structures [1,2]. Dental professionals prefer this material as it provides a range of advantages, including aesthetic appearance, resistance to wear, and capacity to adhere to both enamel and dentin [1,2]. However, composite restorations have a survival rate of 5 to 10 years, which is relatively low [3]. A significant factor in the low survival rate can be attributed to the occurrence of polymerization shrinkage stress, which may result in tooth fracture and recurrent caries [4,5]. Polymerization shrinkage stress occurs when monomer chains are polymerized during the polymerization process [6,7]. This leads to the composite material undergoing shrinkage. Consequently, this contributes to the development of residual shrinkage stresses, primarily arising from the bonding constraints at the interface between the restoration and the tooth [8,9,10]. The stress resulting from the polymerization shrinkage could potentially cause detachment at the tooth–restoration interface, resulting in the formation of marginal gaps and micro-cracks [4,11]. The formation of marginal gaps can result in recurrent caries, which are more likely to happen if no bioactive material is used [12,13].

Several attempts have been employed to minimize shrinkage stress, and one of them involves utilizing resin systems with distinct polymerization characteristics, such as silorane resin-based [14,15], epoxy resin-based [16], and step-growth thiolene resin-based [17]. In addition, the utilization of epoxy oligomers or polymeric nanogel fillers has demonstrated efficacy in mitigating shrinkage stress [18,19]. A novel low-shrinkage-stress (L.S.S.) resin has been recently developed, which is based on ether-based triethylene glycol divinylbenzyl ether (TEG-DVBE) and urethane dimethacrylate (UDMA) [20]. As a result of this formulation, the polymerization rate slowed down, giving the composite more time to achieve its stiffness stage [20]. By utilizing this method, stress relaxation was achieved, and the accumulation of excessive contraction stress was prevented [20]. In addition, UDMA enhanced the resistance of the composite to hydrolysis caused by saliva, whereas TEG-DVBE provided resistance to esterase degradation and other forms of hydrolytic challenges [20]. Another study involved the integration of this novel L.S.S. resin into a dental adhesive material. This particular adhesive exhibited reduced water sorption and solubility, as well as the formation of thicker hybrid layers and more resin tags in comparison to control adhesives [20].

One way to improve the durability of resin-derived materials is by utilizing bioactive agents in their formulations [21,22]. Quaternary ammonium compounds have been widely used to reduce bacterial growth on treated surfaces such as medical devices and textiles [23,24,25,26]. Quaternary ammonium methacrylates (QAMs) are antibacterial monomers that have shown encouraging outcomes for potential clinical use, particularly in inhibiting dental biofilm [21,27,28]. Monomers derived from QAMs, such as dimethylaminohexadecyl methacrylate (DMAHDM) and dimethylaminododecyl methacrylate (DMADDM), have demonstrated strong antibacterial effects against dental biofilm [21]. However, the high viscosity of DMAHDM may pose a challenge when attempting to mix it with other resin monomers. On the other hand, DMADDM is more easily mixed and also possesses contact-killing antimicrobial properties that can lead to bacterial death. Attempts have been made to incorporate DMADDM into dental materials, such as denture base materials, coating agents for dental implants, and resin-based dental composites to develop a bioactive dental material [21,29,30]. A recent study has shown that adding 3% DMADDM to an L.S.S. composite resulted in a potent antibacterial activity against *Streptococcus mutans* (*S. mutans*) biofilms while retaining outstanding mechanical qualities, low polymerization stresses, and an outstanding degree of conversion [31].

Remineralizing fillers, such as nanoparticles of calcium fluoride nanoparticles (nCaF_2_) or amorphous calcium phosphate (NACP), are another strategy for imparting bioactivity in resin-based materials. Composites containing these fillers may change the dynamic process of remineralization/demineralization toward remineralization by releasing high levels of remineralizing ions [32,33,34]. nCaF_2_ promotes high fluoride (F) ion release, promoting remineralization and reducing bacterial acid production, while NACP has been demonstrated to protect enamel hardness and marginal dentin against demineralization [32,33,35]. Such an approach has the potential to reduce demineralization and improve the durability of resin-based materials. A literature search, however, showed no reports on integrating nCaF_2_ or NACP with DMADDM into the L.S.S. nanocomposite.

Currently, there is a rising inclination towards the creation of dental restorations that have better durability. One way to achieve this is by developing bioactive nanocomposites with low-polymerization stress. This approach holds the potential to improve the marginal seal and inhibit the onset of secondary caries [33,36,37]. Earlier research found that utilizing a composite with low polymerization shrinkage stress may improve sealing performance, perhaps preventing secondary caries formation [36,38]. Furthermore, the addition of DMADDM with resin-based materials has demonstrated remarkable antibiofilm activity while not adversely affecting the mechanical properties [31]. Another approach to inhibiting the progression of secondary caries is incorporating nCaF_2_ and NACP into resin-based materials, which have demonstrated promising effects in remineralizing tooth structure [32,39]. A previous investigation explored the incorporation of NACP with L.S.S. composite, which provides a high release of phosphate and calcium ions over 35 days [33]. However, to date, there has been no report on the antibacterial activity, mechanical properties, and cytocompatibility of L.S.S. nanocomposite with the incorporation of DMADDM with nCaF_2_ or NACP.

Consequently, the objective of this study was to explore how the incorporation of DMADDM with nCaF_2_ or NACP impacts the cytocompatibility, antibacterial properties, and mechanical properties of L.S.S. nanocomposites. The following hypotheses were tested. (1) The combination of DMADDM with nCaF_2_ or NACP would not impair the mechanical characteristics of the L.S.S. nanocomposite. (2) The novel nanocomposite with DMADDM and nCaF_2_ or NACP would greatly reduce the biofilm viability, lactic acid synthesis, and metabolic function. (3) The viability of human gingival fibroblast and dental pulp stem cells would not be negatively impacted by the new L.S.S. nanocomposites.

## 2. Materials and Methods

### 2.1. Synthesis of a Nanocomposites

The resin matrix for the L.S.S. nanocomposite, referred to as “UV” resin, was created using 55.8% UDMA and 44.2% of TEG-DVBE (all mass %), which was based on previous studies [31]. Photoinitiators consisting of 0.8% of 4-N, N-dimethylaminobenzoate (4EDMAB; Millipore Sigma) and 0.2% camphorquinone (CQ, Milli-pore Sigma, Burlington, MA, USA) were incorporated.

The Menschutkin reaction was utilized to synthesize DMADDM, which involved combining tertiary amines with organo-halides [40]. To produce DMADDM, a mixture of 10 mmol of 1-bromododecane (BDD) (TCI America, Portland, OR, USA), 10 mmol of 2-(dimethylamino) ethyl methacrylate (DMAEMA, Aldrich, St. Louis, MO, USA), and 3 g of ethanol as the solvent were stirred in a glass vial at 70 °C for 24 h. After the ethanol was evaporated, the resultant DMADDM was a crystal-clear viscous liquid. [31]. DMADDM was added to the UV resin at a final DMADDM mass fraction of 3%.

The nCaF_2_ and NACP were produced using a spray-drying process, as previously reported [32,33]. The nCaF_2_ mean particle size was approximately 32 nm, and the NACP mean particle size was about 116 nm [32,33]. The filler level of nCaF_2_ and NACP was 20% mass fraction in the final composite to preserve strong mechanical characteristics and release a high amount of F, Ca, and P ions for remineralization. Furthermore, at a mass fraction of 45%, silanized barium boroaluminosilicate glass particles (Dentsply Sirona, Milford, DE, USA) were utilized to provide mechanical reinforcement. The total filler level of 65% was chosen based on preliminary experiments to ensure good handling properties. In addition, Heliomolar (Ivoclar, Mississauga, ON, Canada) was selected as a commercial comparison control composite since it comprises 66.7 wt% nano-silica and ytterbium trifluoride and could release fluoride [31]. Six composites were evaluated:

1. Heliomolar composite (designated as “Commercial Control composite”);
2. Experimental composite: 35% UV + 65% glass (designated as “Experimental Control Composite”);
3. 35% UV + 20% NACP + 45% glass (designated as “NACP Nanocomposite”);
4. 32% UV + 3% DMADDM + 20% NACP + 45% glass (designated as “NACP+DMADDM Nanocomposite”);
5. 35% UV + 20% nCaF_2_ + 45% glass (designated as “nCaF_2_ Nanocomposite”);
6. 32% UV + 3% DMADDM + 20% nCaF_2_ + 45% glass (designated as “nCaF_2_+DMADDM Nanocomposite”).

### 2.2. Mechanical Properties

To test the mechanical properties of the composite, a stainless-steel mold with specific dimensions of 2 × 2 × 25 mm^3^ was utilized to create the specimens, which were covered with mylar strips on both sides [31,41]. The samples were cured for 1 min (1200 mW/cm^2^, Labolight, DUO, GC, Tokyo, Japan) [31]. A computer-controlled Universal Testing Machine (Insight 1, MTS, Cary, NC, USA) was used to conduct a three-point flexural test to measure the flexural strength and elastic modulus (*n* = 6), following previous studies [31,42].

### 2.3. Samples Preparation for S. mutans Biofilm Testing

Disk-shaped composites were created with a diameter of 9 mm and a thickness of 2 mm. The surfaces of each sample were cured for 60 **s** (Labolight DUO, GC America, Alsip, IL, USA) and incubated in a 37 °C incubator for 24 h [31]. Next, the samples were placed in distilled water and agitated at 100 rpm for 1 h to eliminate any remaining uncured monomer [31]. The composite disks (*n* = 6) for each group were sterilized using ethylene oxide (Anprolene AN 74i, Andersen, Haw River, NC, USA). To ensure the complete release of entrapped ethylene oxide, the samples were degassed for seven days, following the manufacturer’s guidelines.

### 2.4. Inoculation of S. mutans and Biofilm Formation

Approval was obtained from the University of Maryland Baltimore Institutional Review Board to use bacterial species in the study. *S. mutans* (UA159) was selected as it is one of the organisms commonly associated with dental caries [31]. *S. mutans* was cultured overnight (16–18 h) in brain heart infusion (BHI) broth (Sigma-Aldrich, St. Louis, MO, USA) at 37 °C with 5% CO_2_ for all biofilm assays [31,43]. To adjust the inoculum to 10^7^ colony-forming unit counts CFU/mL, based on the standard curve of OD_600nm_ versus the CFU/mL graph, a spectrophotometer (Genesys 10S, Thermo Scientific, Waltham, MA, USA) was used [31]. The composite samples were placed in the well of 24-well plates, immersed with a 1.5 mL BHI culture medium with 2% sucrose (wt/vol), and incubated for 24 h. The composite samples were then transferred to 24-well plates, immersed with 1.5 mL of fresh medium with sucrose, and incubated for another 24 h. According to a previous study, incubation for 48 h was sufficient to form relatively mature biofilms on composite disks [31].

### 2.5. Examining S. mutans Biofilms Using Scanning Electron Microscopy (SEM)

At 48 h, biofilms on composites were washed with phosphate-buffered saline (PBS) and treated with 1% glutaraldehyde at 4 °C overnight. Subsequently, the composite samples were rinsed with PBS and dehydrated using a series of ethanol solutions. Next, the samples were rinsed with hexamethyldisilazane and allowed to dry overnight. The surface of the samples was sputter-coated with platinum. SEM (Quanta 200, FEI Company, Hillsboro, OR, USA) was utilized to visualize biofilms on composites [44].

### 2.6. S. mutans Biofilm Colony-Forming Units (CFU)

The composite samples containing 48-h biofilms were moved to a fresh plate containing PBS, and the biofilms were harvested by scraping and sonicating/vortexing (FS-30, Fisher, Pittsburg, PA, USA) [33]. The bacterial suspensions were diluted serially (10^1^–10^6^-fold) and spread onto BHI agar plates, which were then incubated for 48 h at 37 °C with 5% CO_2_. The colony number was counted, and the biofilm (CFU) was determined [31]. The CFU experiment was performed in triplicate.

### 2.7. Metabolic Activity of S. mutans Biofilms

Biofilm metabolic activity was assessed using a colorimetric assay involving 3-[4,5-dimethylthiazol-2-yl]-2,5-diphenyltetrazolium bromide (MTT) [31]. The composite samples with biofilms were placed in a fresh 24-well plate and treated with 1 mL of MTT dye (0.5 mg/mL MTT in PBS). The plate was then incubated at 37 °C in a 5% CO_2_ environment. After 1 h, the samples were transferred to another plate containing 1 mL of dimethyl sulfoxide (DMSO) and kept in the dark at room temperature for 20 min to dissolve the formazan crystals [31]. To determine the absorbance, 200 μL of the DMSO solution was collected from each specimen and transferred to a 96-well plate. The absorbance was measured at 540 nm using a microplate reader (SpectraMax M5, Molecular Devices, Sunnyvale, CA, USA). A higher absorbance indicates a higher concentration of formazan, indicating increased metabolic activity of the biofilm on the disk [31]. The metabolic activity experiment was performed in triplicate.

### 2.8. Lactic Acid Production by S. mutans Biofilms

The composites with biofilms at 48 h were moved to 24-well plates containing buffered peptone water (BPW, Aldrich, St. Louis, MO, USA) with 0.2% sucrose and incubated for 3 h at 37 °C in 5% CO_2_ [31]. To determine lactate concentrations in BPW, the optical density was measured at 340 nm using a microplate reader (Spectra-Max M5) via lactate dehydrogenase enzymatic assay, following previously described methods [31]. The lactic acid production experiment was performed in triplicate.

### 2.9. Cytotoxicity of Human Gingival Fibroblasts and Dental Pulp Stem Cells

Human gingival fibroblast (HGF, P10866, Innoprot, Bizkaia, Spain) and dental pulp stem cells (DPSC, PT-5025, Lonza, Basel, Switzerland) were utilized to evaluate cytotoxicity. The fibroblast medium (Sciencell Research Laboratories, Carlsbad, CA, USA), consisting of 1 wt.% fibroblast growth supplement, 2 wt.% fetal bovine serum, 100 IU/mL streptomycin, and 100 IU/mL penicillin, was used to culture HGF (passage 7) [45]. DPSCs (passage 6) were cultured in DPSC basal medium (DPSC, PT-5025, Lonza, Basel, Switzerland) supplemented with 2 mM L-glutamine, 2 wt.% fetal bovine serum, 100 mM ascorbic acid solution, GA-1000, 100 IU/mL streptomycin, and 100 IU/mL penicillin [46]. Cell seeding was performed when the viability of the cells had exceeded 90%.

In a 96-well plate with the corresponding medium, cells were seeded at a density of 5000 cells per well and incubated for 24 h at 37 °C with 5% CO_2_ [45,46,47]. Disk-shaped composite samples (*n* = 3, diameter of 4 mm, and thickness of 1 mm) were sterilized in the same manner as previously described for antibacterial testing. After that, the samples were put in 4 mL of medium and left at 37 °C for 24 h, yielding a surface area to solution ratio of 0.63 cm^2^/mL, which falls within the required range of 0.5–6 cm^2^/mL as per ISO 10993-12:2021 [48]. Next, 100 mL of each sample’s original extracts were added to cultured cells and incubated for 24 h at 37 °C with 5% CO_2_. After incubation, each well received 10 mL of cell counting kit-8 (CCK-8, Dojindo, Rockville, MD, USA) and was incubated for 2 h under the same conditions [49]. The absorbance at 450 nm was used to evaluate the degree of cellular dehydrogenase activity in the culture medium, while the control group consisted of cells cultured in media alone [49]. The cell viability experiment was performed in triplicate.

### 2.10. Statistics

The statistical analyses, including normality verification and power analysis, were performed using Sigma Plot (SYSTAT, Chicago, IL, USA). One-way analyses of variance (ANOVA) and Tukey’s comparison tests were performed to determine the significant differences between groups in load-bearing properties, antibacterial effects, and cell viability. The data were deemed statistically significant at a *p*-value of below 0.05.

## 3. Results

### 3.1. Mechanical Properties

**Figure 1:**
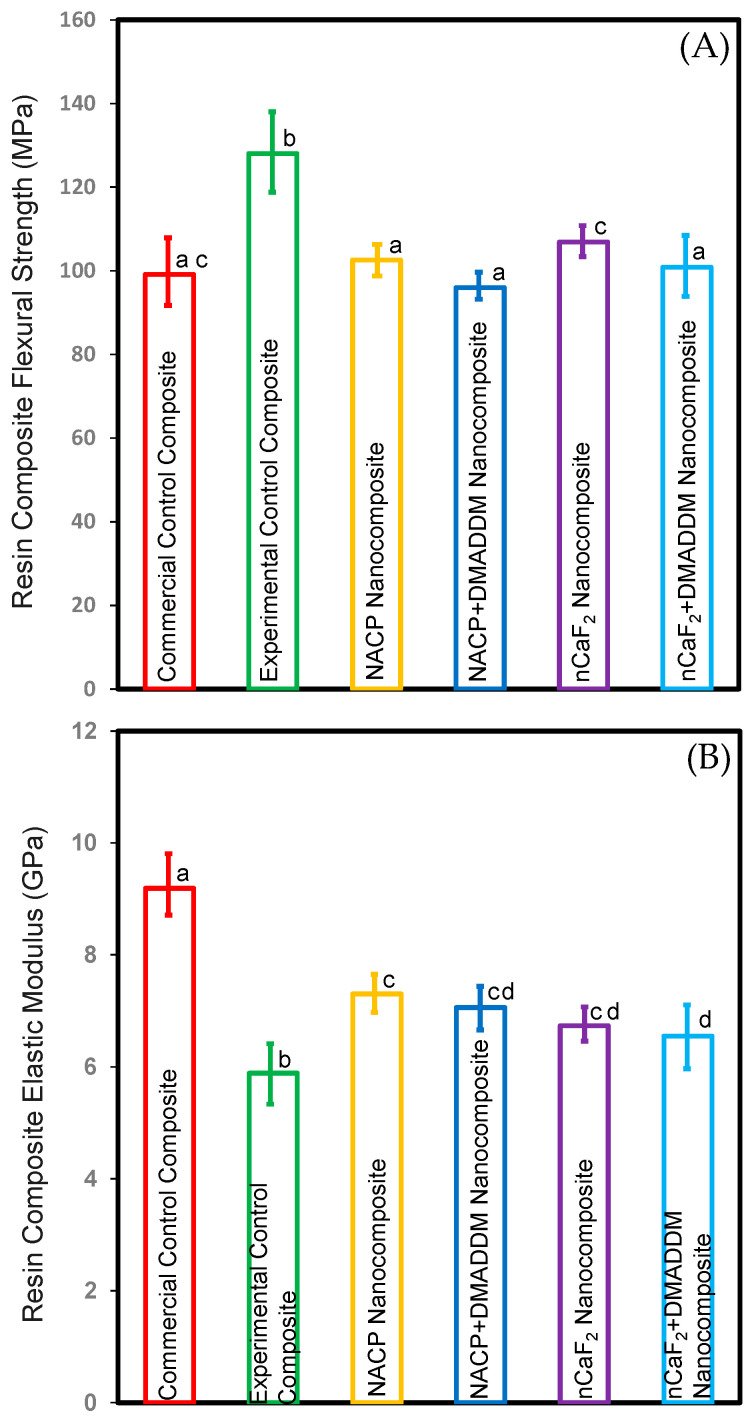
Composite mechanical properties: (**A**) flexural strength, and (**B**) elastic modulus (mean ± sd; *n* = 6). Incorporating DMADDM with nCaF_2_ or NACP into an L.S.S. nanocomposite did not compromise the flexural strength compared to commercial control composite (*p* > 0.05). Incorporating nCaF_2_ or NACP with DMADDM did not compromise the elastic modulus compared to the experimental control composite (*p* < 0.05). Dissimilar letters represent values that are significantly different from each other (*p* < 0.05).

Figure 1 depicts the flexural strengths and modulus of elasticity of the composites (mean ± sd; *n* = 6): (A) flexural strength and (B) elastic modulus. The incorporation of nCaF_2_ and NACP with and without DMADDM in the L.S.S. nanocomposite resulted in flexural strength values comparable to the commercial control composite (*p* > 0.05). These outcomes demonstrate that our novel formulations did not affect flexural strength.

The commercial control composite had much higher elastic modulus values than all other testing groups (*p* < 0.05). However, these results demonstrated that incorporating nCaF_2_ and NACP with and without DMADDM into the L.S.S. nanocomposite increased the elastic modulus compared to the experimental control composite (*p* < 0.05).

### 3.2. Examination of S. mutans Biofilms Using SEM

**Figure 2:**
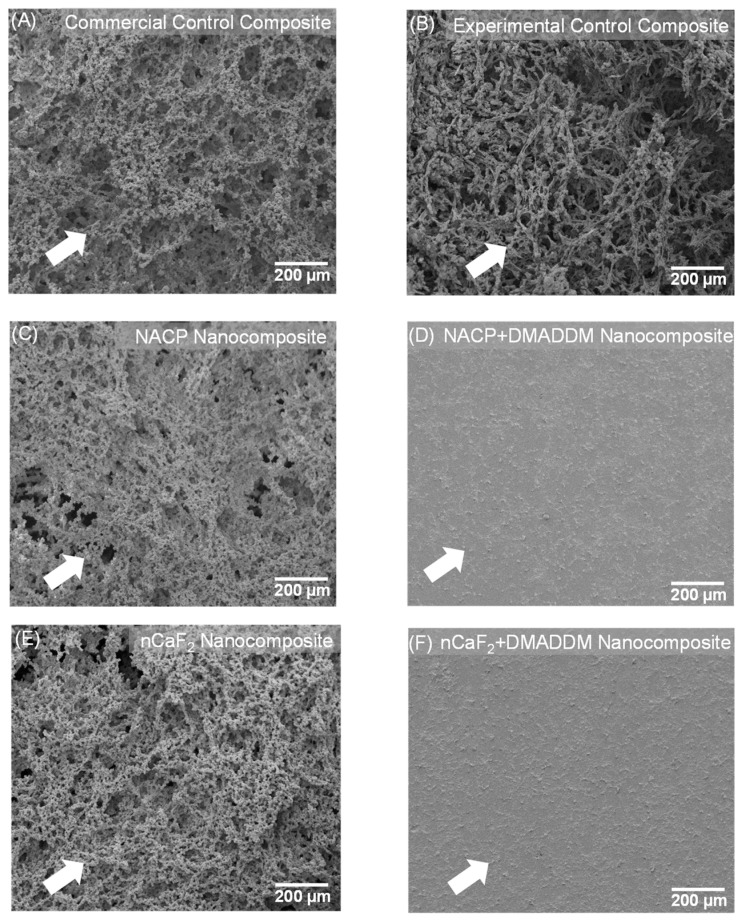
SEM examination of biofilms on composites at 2 days (*n* = 6). (**A**) Commercial control composite. (**B**) Experimental control composite. (**C**) NACP nanocomposite. (**D**) NACP+DMADDM nanocomposite. (**E**) nCaF_2_ nanocomposite. (**F**) nCaF_2_+DMADDM nanocomposite. DMADDM greatly reduced *S. mutans* biofilm growth on composites. In comparison, NACP nanocomposite, nCaF_2_ nanocomposite, and control composite groups were covered with biofilms.

Figure 2 displays the SEM findings after 48 h of *S. mutans* biofilms (*n* = 6). The nCaF_2_ nanocomposite, NACP nanocomposite, and control composite groups demonstrated significant biofilm formation after 48 h. Conversely, our result showed that incorporating DMADDM with either nCaF_2_ or NACP into the L.S.S. resin matrix resulted in minimal bacterial biofilm formation.

### 3.3. S. mutans Biofilm CFU

**Figure 3:**
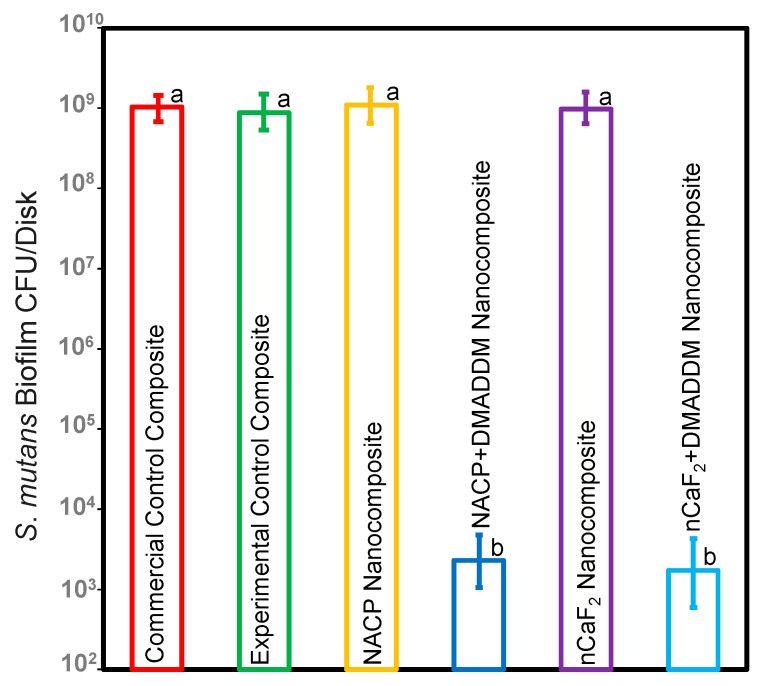
CFU of *S. mutans* biofilms on composites (mean ± sd; *n* = 6). The nCaF_2_ nanocomposite, NACP nanocomposite, and control composite had the highest biofilm growth. DMADDM inhibited *S. mutans* biofilm growth by 6 logs compared to commercial control composite (*p* < 0.05). Dissimilar letters represent values that are significantly different from each other (*p* < 0.05).

Figure 3 depicts the CFU findings of the *S. mutans* biofilm (mean ± sd; *n* = 6). Incorporating nCaF_2_ and NACP into an L.S.S. nanocomposite showed no antibacterial effect without incorporating DMADDM. However, our results demonstrated that incorporating DMADDM with either nCaF_2_ or NACP into an L.S.S. nanocomposite significantly decreased the CFU count for *S. mutans* biofilm by 6 logs when compared to other groups (*p* < 0.05).

### 3.4. Metabolic Function of S. mutans Biofilms

**Figure 4:**
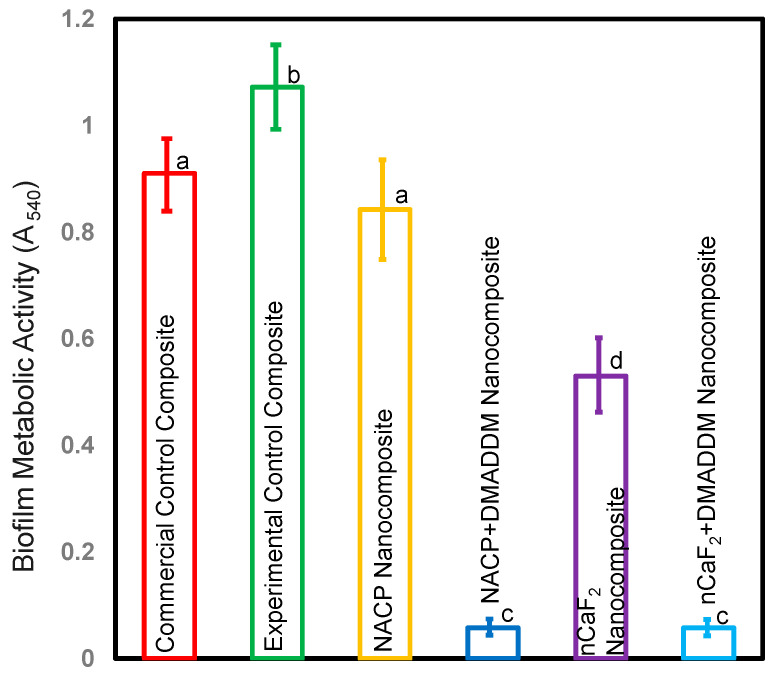
Metabolic activity of biofilms on composites after two days (mean ± sd; *n* = 6). Incorporating DMADDM with either nCaF_2_ or NACP into an L.S.S. nanocomposite diminished the metabolic function of *S. mutans* by over 90% compared with other groups (*p* < 0.05). Different letters represent data that are significantly different from each other (*p* < 0.05).

Figure 4 depicts the bacterial metabolic function of biofilms on composite samples after two days (mean ± sd; *n* = 6). Integrating DMADDM with either nCaF_2_ or NACP into an L.S.S. nanocomposite considerably lowered metabolic activities by roughly 90% when compared to other groups (*p* < 0.05). However, there was no statistically significant variation in metabolic activity between these groups (*p* > 0.05).

### 3.5. Production of Lactic Acid by S. mutans Biofilms

**Figure 5:**
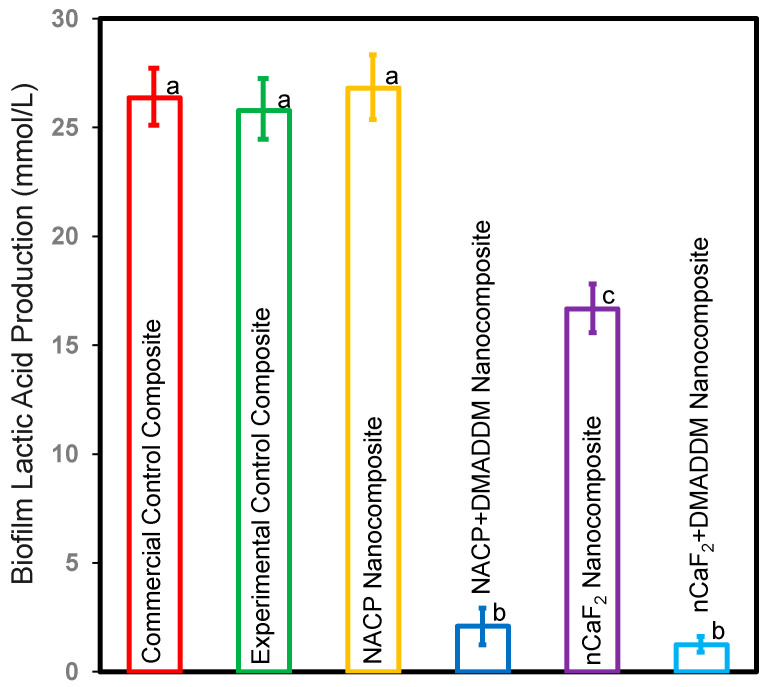
Lactic acid production of *S. mutans* biofilms on composites (mean ± sd; *n* = 6). The NACP nanocomposite and control composite groups had the highest lactic acid production (*p* < 0.05). The nCaF_2_ nanocomposite reduced lactic acid production compared to control groups (*p* < 0.05). However, incorporating DMADDM diminished lactic acid production by over 92% compared to other groups (*p* < 0.05). Different letters represent data that are significantly different from each other (*p* < 0.05).

Figure 5 depicts the lactic acid production of *S. mutans* biofilms adhered to composites (mean ± sd; *n* = 6). The experimental control composite, commercial control composite, and NACP nanocomposite groups had the highest acid production (*p* < 0.5). Adding nCaF_2_ reduced acid generation compared to the commercial control composite (*p* < 0.5). Nevertheless, adding DMADDM into an L.S.S. nanocomposite containing either nCaF_2_ or NACP dramatically decreased lactic acid generation by more than 92% when compared to other groups (*p* < 0.5). Our results revealed that the incorporation of DMADDM demonstrated the highest biofilm reduction.

### 3.6. Cytotoxicity Test

**Figure 6:**
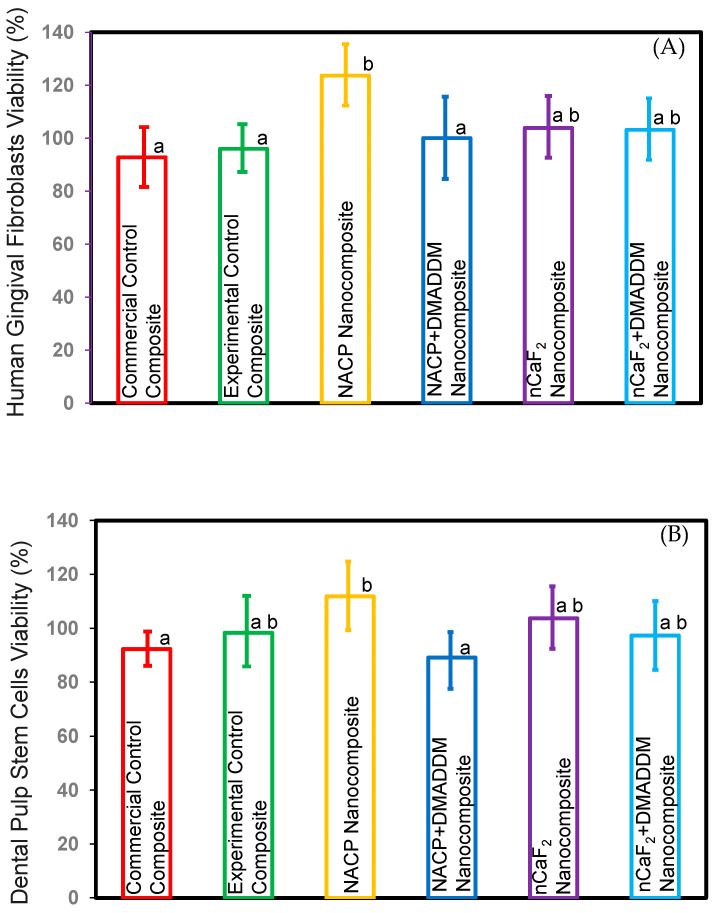
Cell viability of composites. (**A**) HGF, (**B**) DPSCs (mean ± sd; *n* = 3 × 3). These results showed that the NACP nanocomposite had a significant increase in viability compared to the commercial control composite (*p* < 0.05). All the other novel nanocomposite groups showed comparable viability to commercial control composite (*p* > 0.05). Different letters represent data that are significantly different from each other (*p* < 0.05).

The viability toward the newly developed L.S.S. nanocomposite: (A) HGF, (B) DPSCs (mean ± sd; 3 experimental repeats, each with *n* = 3 technical replicates) plots in Figure 6. The NACP nanocomposite group demonstrated significantly increased HGF and DPSCs cell viability compared to the commercial control composite (*p* < 0.5). However, all other novel nanocomposite groups showed comparable cell viability to the commercial control composite (*p* > 0.5).

## 4. Discussion

For the first time, the present work explored the effect of incorporating nCaF_2_ and NACP with and without DMADDM into an L.S.S. nanocomposite. Incorporating nCaF_2_, NACP, and DMADDM into an L.S.S. nanocomposite may potentially decrease polymerization shrinkage stress, marginal leakage, and secondary caries [33,36]. As a result, the clinical durability of composite restorations could be improved [36]. In the current study, we studied the antibacterial property and cytocompatibility of a novel L.S.S. nanocomposite, incorporating 20% nCaF_2_ or 20% NACP, with and without 3% DMADDM. The capability of L.S.S. nanocomposite to suppress *S. mutans* biofilm without negatively influencing mechanical characteristics and cytocompatibility was achieved, and the study hypotheses were proved. Incorporating DMADDM with nCaF_2_ or NACP into the L.S.S. nanocomposite resulted in a significant reduction of *S. mutans* biofilm growth compared to the control groups. This reduction in biofilm growth was observed while maintaining mechanical properties that aligned with clinical standards. Furthermore, the addition of DMADDM with nCaF_2_ or NACP was associated with a decrease in metabolic and lactic acid production activity of *S. mutans* biofilm, suggesting potential for preventing secondary caries.

Several attempts have been made to develop a low-shrinkage composite, such as the use of epoxy oligomers fillers [19], polymeric nanogel fillers [18], the epoxy resin-base [16], silorane resin-base [14], and step-growth thiolene resin-base [17]. However, these approaches negatively affect the mechanical properties without improving the margin integrity [50]. Recently, UDMA and TEGDVBE monomers were combined to create a composite with minimal shrinkage stress [33]. This composite demonstrated low polymerization stress while maintaining a high degree of conversion and excellent mechanical properties [33]. In addition, several attempts have been made to create a bioactive L.S.S. composite by adding DMAHDM to the L.S.S. resin. However, the lengthy chain of DMAHDM can elevate the viscosity of the resin, which can decrease the degree of conversion and filler load that can be incorporated into the composite [51]. Moreover, the antibacterial outcomes of adding DMAHDM to the resin composite may not be consistent due to the molecule’s long chain length. This extended chain may decrease the charge density of quaternary ammonium at the surface, which could impact the composite’s antibacterial properties [51]. Recently, a bioactive L.S.S. incorporating DMADDM demonstrated a substantial reduction of 46% in polymerization shrinkage stresses in comparison to a conventional resin-based control composite [31]. In addition, the incorporation of 3% DMADDM exhibits potent antibacterial properties without negatively impacting the degree of polymerization conversion and mechanical properties [31]. However, no report has investigated the effect of incorporating nCaF_2_ or NACP with DMADDM into an L.S.S. nanocomposite.

One of the strategies to impart bioactivity in resin-based materials is the incorporation of remineralizing fillers in the composite system [52,53,54]. Nanocomposites containing nCaF_2_ or NACP can alter the dynamic process of remineralization/demineralization in favor of remineralization [33,55]. This is achieved by releasing a significant amount of remineralizing ions [33,55]. The current research involved the incorporation of nCaF_2_ and NACP particles into an antibacterial nanocomposite that has low polymerization stress. The purpose of this was to produce a smart material that effectively interacts with the oral environment. Composites containing nCaF_2_ and NACP could potentially help to raise the pH and modify the microenvironment around the dental plaque [56]. Furthermore, incorporating nCaF_2_ and NACP into the composite may provide another potential benefit: the ability to recharge with Ca, P, and F ions, resulting in an extended release of these ions over time [44,56]. In addition, the incorporation of NACP into an L.S.S. nanocomposite demonstrated good mechanical properties even after 20,000 thermal cycles [57]. Another study found that incorporating 20% nCaF_2_ into the composite demonstrated excellent mechanical properties even after 10^5^ thermal cycles [32]. The current study was designed to explore the antibacterial property and cytocompatibility of an L.S.S. nanocomposite containing nCaF_2_ or NACP with or without DMADDM. Incorporating nCaF_2_ and NACP with and without DMADDM achieved comparable flexural strength to commercial control composite (*p* > 0.05). Conversely, the newly developed L.S.S. composite exhibited a lower modulus of elasticity when compared to the commercial control composite (*p* < 0.05). We hypothesize that one possible reason for this difference is the variation in the size and type of the filler particles used in these composites [31]. Additionally, it is possible that the different resin matrices used in the commercial control composite could also contribute to the enhancement of its elastic modulus [6,58]. In previous studies, the addition of 15% nCaF_2_ to a conventional composite did not compromise the mechanical characteristics [44,59]. However, in the present study, there was a slight decrease in flexural strength at 20% nCaF_2_ in the L.S.S. nanocomposite when compared to the experimental control. This difference was likely due to the relatively lower concentration of silane-treated barium boroaluminosilicate glass particles for reinforcement in the current study, which could decrease the mechanical properties.

Dental plaque may have a negative impact on the durability of dental restorations through the development of recurrent caries and the production of enzymes that could cause material degradation [60,61,62]. Therefore, developing a novel bioactive material that possesses remineralization and antibacterial properties, as well as the ability to reduce polymerization stress, could increase the clinical longevity of the composite restoration. In an earlier investigation, chlorhexidine was introduced to a composite with a low-shrinkage-stress resin system to develop an antibacterial effect [63]. However, they found it has a short-time effect [63]. Another effort was made to use zinc oxide and silver nanoparticles as antibacterial additives in resin-based materials [64,65,66]. However, these nanoparticles were observed to detach from the surface of the material relatively quickly, which resulted in increased porosity and a decrease in mechanical strength [65,66]. To address this significant issue, a novel approach was developed, which involved the use of QAMs [21,67,68]. These compounds could be covalently bonded with dental resins through copolymerization, resulting in long-lasting antibacterial effects [21]. The addition of QAMs in resin-based materials exhibited a strong antibacterial impact by employing a contact-killing mechanism [55]. QAMs have a positively charged quaternary amine N^+^ that can attach to the negatively charged membrane of bacterial cells [55]. This alters the balance of essential ions such as Na^+^, Ca^2+^, Mg^2+^, and K^+^ and results in leakage from the cytoplasm, which ultimately causes the bacterial membrane to break down [55]. Earlier research studies have indicated that the antibacterial efficacy of QAMs can be enhanced by increasing their chain length, as this increases their hydrophobicity, thereby enabling them to penetrate the hydrophobic bacterial cell membrane more effectively [21,67]. Furthermore, a previous study showed that a resin containing DMADDM maintained strong antibacterial effects even after six months of water aging while still possessing good mechanical properties [69]. The current study thoroughly investigated the effects of incorporating nCaF_2_ or NACP with or without DMADDM into an L.S.S. nanocomposite. Incorporating DMADDM with nCaF_2_ or NACP into an L.S.S. nanocomposite demonstrated significant antibacterial activity against *S. mutans* biofilm. This formulation significantly diminished the CFU count by 6 logs compared to the commercial control composite (*p* < 0.05). Furthermore, our findings demonstrated that the addition of DMADDM in combination with nCaF_2_ or NACP resulted in a reduction of over 85% in metabolic activities and lactic acid production compared to the commercial control composite. Moreover, SEM images validate the existence of *S. mutans* biofilms on the surfaces of nCaF_2_ nanocomposite, NACP nanocomposite, and control composite groups. Conversely, the incorporation of DMADDM along with nCaF_2_ or NACP resulted in a decrease in the attachment of bacteria to the surface. Nevertheless, our findings indicated that there was no significant difference in antibacterial impact when integrating DMADDM to either nCaF_2_ or NACP against *S. mutans* biofilms (*p* > 0.05). This formulation could be beneficial in various applications due to its L.S.S. and excellent bioactivity. For example, it could serve as a base for bulk dental restorations, as a composite restoration in Class V cases, and as a flowable composite for pits and fissures, etc. Additionally, its antibacterial properties could aid in reducing plaque accumulation, especially in patients with poor oral hygiene, potentially leading to a decrease in recurrent caries and gingival inflammation [31,70].

Despite the potential of the novel L.S.S. nanocomposite demonstrated in this study, it is crucial for the material to be non-toxic to human cells. This research represents the first investigation interested in the cytotoxic properties of this particular L.S.S. nanocomposite with antibacterial and remineralizing capabilities. This was achieved by utilizing the extract obtained directly from the cured disks through immersion in fibroblast medium and DPSC basal medium at 37 °C for 24 h. All the evaluated groups were classified as non-toxic in line with the recommendations of the International Organization for Standardization (ISO), as their cell viability measurements exceeded 75% [71]. As per the present study, it was found that the nCaF_2_ and NACP groups exhibited an improvement in cell viability when compared to the commercial control composite. The results from the present study are consistent with previous research that has shown that using calcium phosphate cement (CPC) for direct pulp capping may facilitate the differentiation of human dental pulp cells (HDPCs) into odontoblasts [72]. In addition, a previous study found that the release of calcium ions is considered crucial in the process of mineralization as it helps promote cellular migration and differentiation [73]. Furthermore, another study found that the use of nanocomposites and adhesives containing NACP and DMADDM resulted in lower levels of inflammatory response and increased tertiary dentin formation in a rat tooth cavity model [30]. These results suggest that using nCaF_2_, NACP, and DMADDM in restorative materials may have promising clinical applications in promoting pulp healing and tissue regeneration. The L.S.S. nanocomposite demonstrated strong antibacterial properties while maintaining excellent biocompatibility against HGF and DPSCs. However, further studies are needed to investigate the biocompatibility of this novel nanocomposite in vivo. These studies could provide valuable insights into the clinical performance of the composite and its potential use in restorative dentistry.

Integrating DMADDM with nCaF_2_ or NACP into the L.S.S. nanocomposite resulted in potent antibacterial characteristics without negatively impacting the load-bearing properties. The addition of DMADDM decreased biofilm CFU counts by 6 orders of magnitude compared to commercial composite. Furthermore, the new formulations showed excellent cytocompatibility. Therefore, developing an L.S.S. nanocomposite with antibacterial and remineralization properties can potentially address some of the major challenges associated with traditional composite restorations, such as secondary caries and restoration failure. Incorporating antibacterial agents together with remineralizing agents into an L.S.S. nanocomposite could reduce microleakage, inhibit bacterial growth, and promote remineralization of the tooth structure. This can lead to improved clinical outcomes and increased longevity of the restoration.

Additional research is required to address several limitations of the current study, including investigating the antibacterial effectiveness of the novel L.S.S. nanocomposite against multispecies biofilms that more closely resemble clinical conditions. Moreover, long-term investigations are still needed to assess the impact of incorporating a combination of nCaF_2_, NACP, and DMADDM into a single formulation on the antibacterial and mechanical properties of the L.S.S. nanocomposite. Furthermore, research is also needed to study the marginal seal of this new L.S.S. nanocomposite as compared to commercial composites [74]. Further studies are also needed to investigate the effects of integrating DMADDM with nCaF_2_ or NACP into the L.S.S. nanocomposite on the composite surface properties [75] and color stability [76].

## 5. Conclusions

This study developed a novel L.S.S. nanocomposite with antibacterial and remineralization capabilities. Incorporating DMADDM provided a potent antibacterial impact while maintaining mechanical properties. The new formulation achieved a reduction of 6-log in biofilm CFU, along with a significant decrease in biofilm lactic acid production and metabolic activity. In addition, the L.S.S. nanocomposite demonstrated excellent biocompatibility against HGF and DPSCs. The new bioactive L.S.S. nanocomposite has promise in various dental restorations to improve marginal integrity by reducing shrinkage stress, protecting tooth structures, and reducing cariogenic biofilms.

## Data Availability

The data presented in this study are available on request from the corresponding author.
